# Pulmonary Leiomyoma in a Post-hysterectomy Patient: A Diagnostic Challenge

**DOI:** 10.7759/cureus.108876

**Published:** 2026-05-15

**Authors:** Ajna S Kumar, Thangaswamy Dhanasekar, Irfan Ismail Ayub, Abdul Majeed Arshad

**Affiliations:** 1 Pulmonology, Sri Ramachandra Institute of Higher Education and Research, Chennai, IND

**Keywords:** benign metastasizing leiomyoma (bml), lung leiomyoma, lung smooth muscle tumor, primary pulmonary leiomyoma, pulmonary benign metastasizing leiomyoma, pulmonary leiomyoma

## Abstract

Pulmonary leiomyoma is a rare benign smooth muscle tumor that may present as a solitary lung mass and mimic malignancy. In women with a prior history of uterine leiomyoma and hysterectomy, the possibility of benign metastasizing leiomyoma (BML) must be considered. We report a case of a middle-aged woman with a prior hysterectomy for uterine leiomyoma who presented with an endobronchial tumor, suspicious for malignancy. The patient underwent lobectomy, and histopathological evaluation confirmed the leiomyoma of the lung. This case highlights the diagnostic dilemma posed by such lesions and underscores the importance of histopathological and immunohistochemical evaluation in establishing the diagnosis.

## Introduction

Uterine leiomyoma is the most common benign tumor of the female genital tract. In rare instances, it demonstrates unusual behavior with extrauterine spread, termed benign metastasizing leiomyoma (BML) [1]. BML is characterized by the presence of histologically benign smooth muscle tumors at distant sites, most commonly the lungs [2].

The true incidence of benign metastasizing leiomyoma remains unknown due to its rarity and the predominance of isolated case reports and small case series in the literature, with fewer than 100 cases reported in the literature worldwide [3].

Pulmonary involvement typically occurs years after hysterectomy or myomectomy performed for uterine leiomyoma, suggesting a metastatic or multifocal process [3]. Although most cases present with multiple bilateral nodules, solitary pulmonary lesions have also been described, making differentiation from primary lung malignancy particularly challenging [4].

Radiologically, these lesions often appear as well-circumscribed nodules or masses and lack specific features to distinguish them from malignant tumors. Consequently, surgical resection is frequently required for definitive diagnosis. We report a rare case of pulmonary leiomyoma presenting as an endobronchial tumor in a woman with a history of hysterectomy for uterine leiomyoma.

## Case presentation

A 58-year-old woman presented with progressively worsening dyspnea and intermittent nonproductive cough of two-month duration. There was no history of smoking, hemoptysis, weight loss, fever, or prior malignancy. Her past medical history was significant for a total abdominal hysterectomy performed three years earlier for a uterine leiomyoma. General physical examination and systemic evaluation were unremarkable, and routine hematological and biochemical investigations were within normal limits. Chest radiography demonstrated the collapse of the left upper lobe, prompting further evaluation with flexible video bronchoscopy.

**Figure 1:**
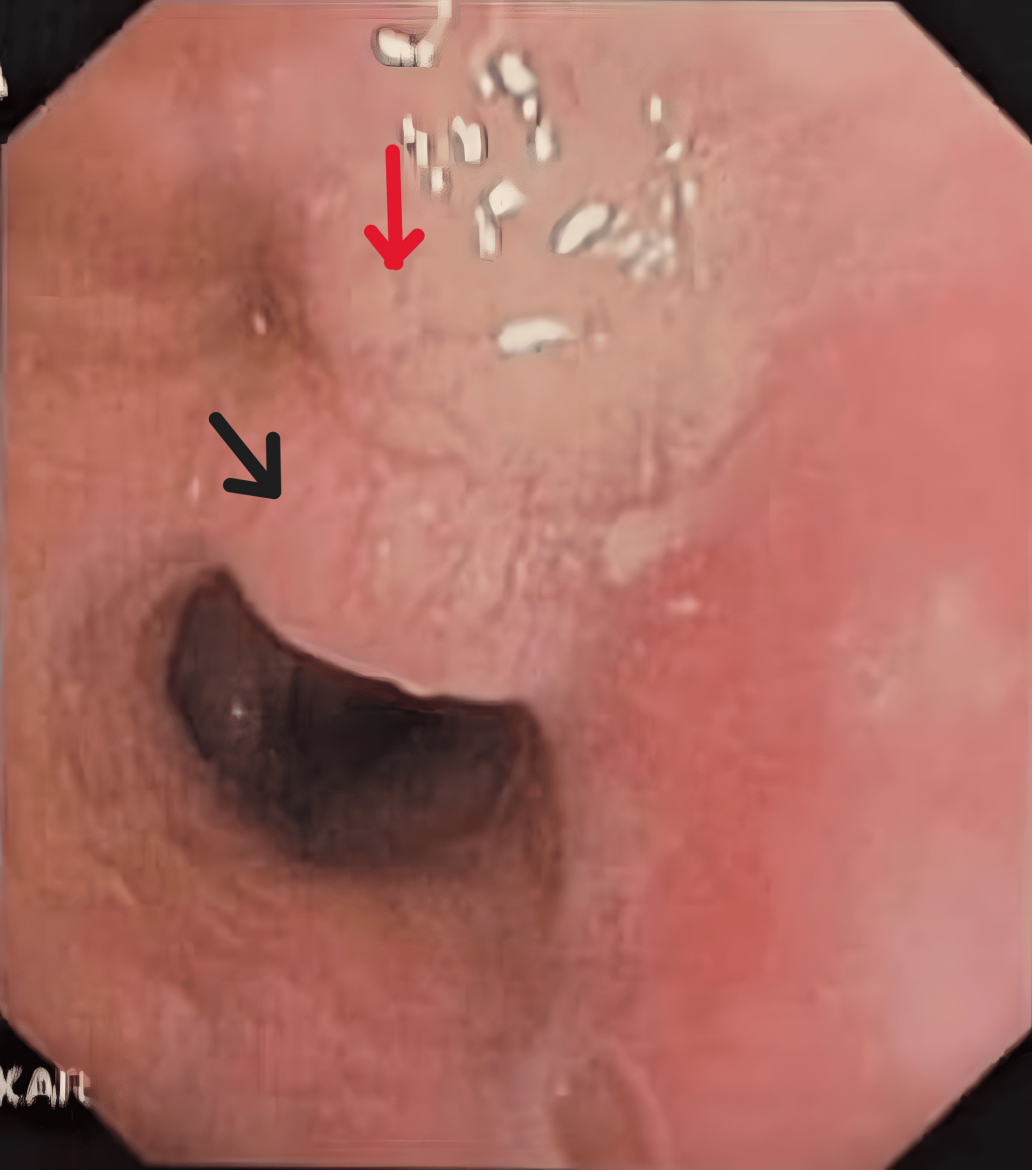
Bronchoscopy image showing a pedunculated tumor in the left upper lobe (red arrow) protruding into the left main bronchus (back arrow)

Bronchoscopic examination revealed a pedunculated endobronchial mass arising from the left upper lobe bronchus and protruding into the left main bronchus (Figure 1), causing near-complete luminal obstruction and preventing the advancement of the bronchoscope into the left lower lobe.

**Figure 2:**
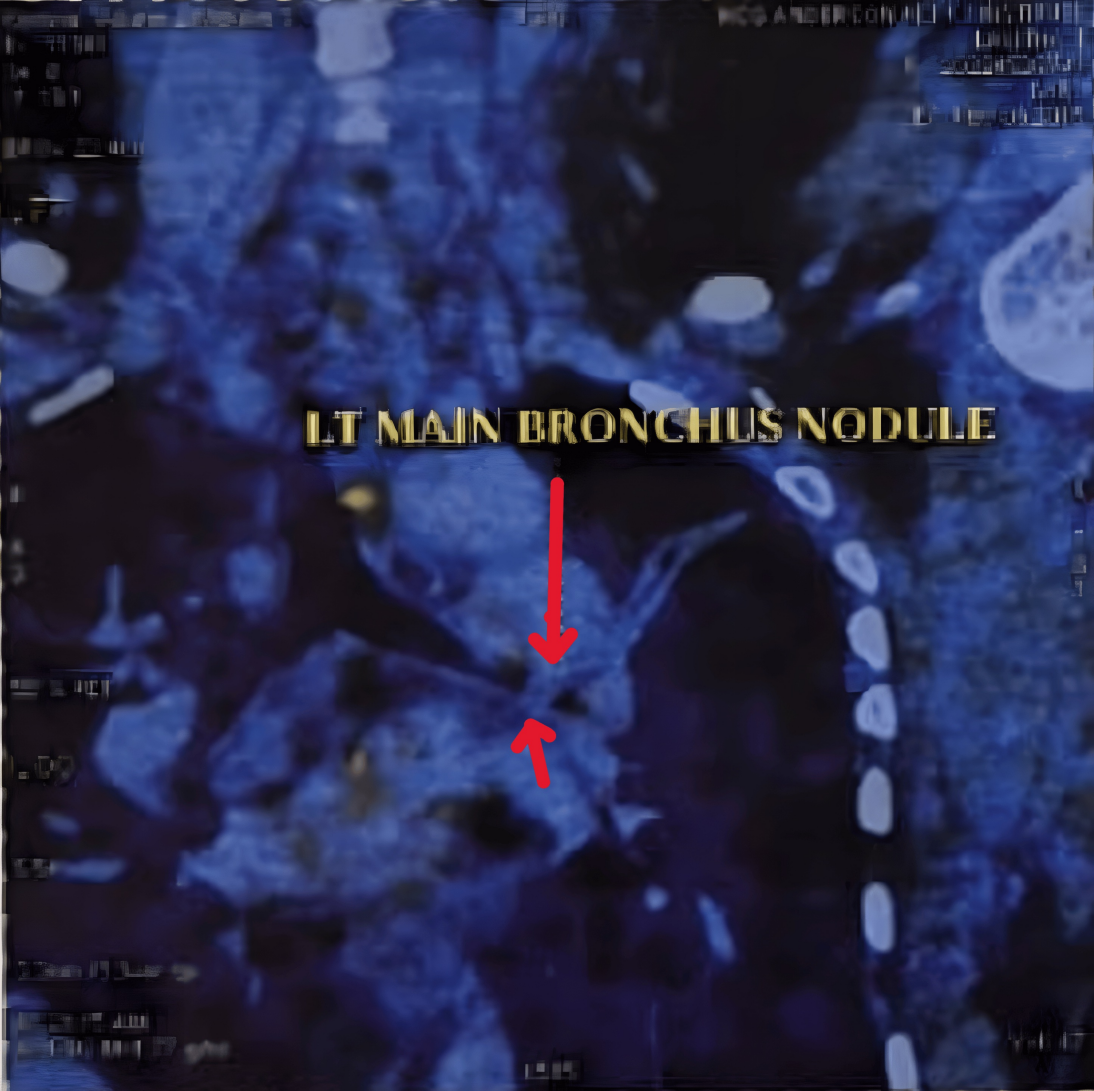
Coronal section of the PET-CT image of the thorax showing a nodule in the left main bronchus (indicated by red arrows) PET-CT: positron emission tomography-computed tomography

**Figure 3:**
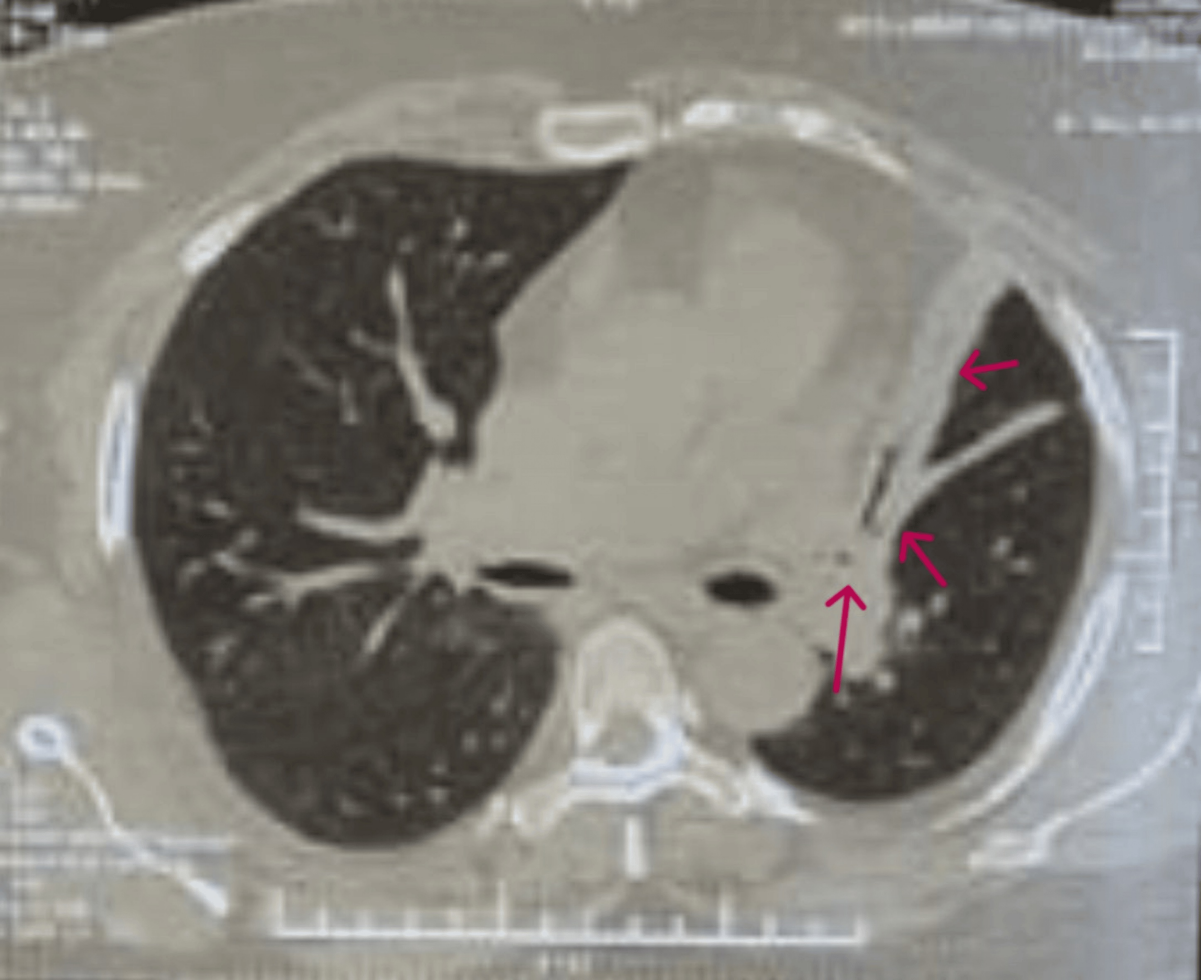
Axial section of the computed tomography of the thorax showing an endoluminal lesion in the left upper lobe with linear atelectasis of the left lingular segment, anterior and posterior segments of the left upper lobe

**Figure 4:**
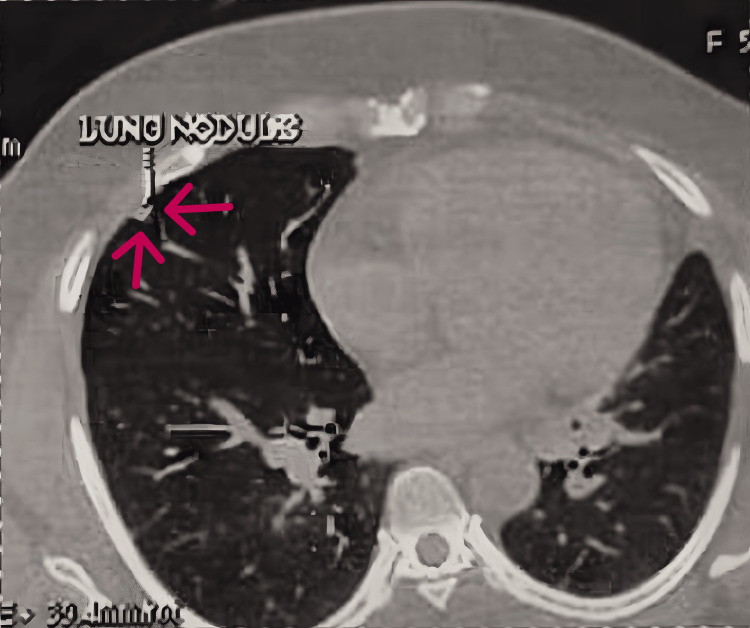
Axial section of the computed tomography of the thorax showing a subpleural nodule (indicated by red arrows) in the right upper lobe

Positron emission tomography-computed tomography (PET-CT) demonstrated a small hypodense endoluminal lesion measuring 7 × 6 mm in the proximal left upper lobe bronchus (Figures 2, 3), along with multiple bilateral subpleural pulmonary nodules (Figure 4) containing specks of calcification, without evidence of mediastinal lymphadenopathy or distant metastasis. The lesion showed poor enhancement with no significant metabolic activity.

In view of the high clinical suspicion for primary bronchogenic malignancy, the patient underwent left upper lobectomy. The histopathological examination of the resected specimen revealed intersecting fascicles of spindle-shaped cells with elongated blunt-ended nuclei and eosinophilic cytoplasm, without cytological atypia, necrosis, or significant mitotic activity. Immunohistochemical analysis showed tumor cell positivity for smooth muscle actin (SMA), desmin, estrogen receptor (ER), and progesterone receptor (PR), while cytokeratin, CD34, and S-100 were negative, confirming the diagnosis of pulmonary leiomyoma. The postoperative course was uneventful, and the patient has remained asymptomatic with no evidence of recurrence during 10 years of follow-up.

## Discussion

Benign metastasizing leiomyoma is a rare condition, with fewer than 100 cases reported in the literature [3]. It predominantly affects women of reproductive or perimenopausal age who have a prior history of uterine leiomyoma, often treated with hysterectomy [1].

The lung is the most common site of involvement, although other sites such as lymph nodes, the bone, and soft tissues have been described [1]. The interval between hysterectomy and pulmonary presentation can range from months to several decades [4].

Radiologically, pulmonary BML (PBML) most commonly presents as multiple bilateral nodules (approximately 87% of cases), while solitary nodules are less common and can closely mimic primary lung carcinoma [4]. In such cases, preoperative differentiation from malignancy is extremely difficult, often necessitating surgical resection. Histologically, pulmonary leiomyomas consist of well-differentiated smooth muscle cells arranged in interlacing fascicles, with minimal atypia and low mitotic activity. Immunohistochemical staining typically shows positivity for smooth muscle markers (SMA and desmin) and hormone receptors, while epithelial and neural markers are negative [1].

The pathogenesis of BML remains controversial. Proposed mechanisms include the hematogenous spread of benign uterine leiomyoma cells or multifocal smooth muscle proliferation influenced by hormonal factors [2]. The expression of estrogen and progesterone receptors in many cases supports a hormone-sensitive nature of the disease.

Management strategies vary depending on presentation. Surgical resection is often performed for solitary lesions for both diagnosis and treatment, as in our case. In patients with multiple lesions, hormonal therapy (e.g., aromatase inhibitors or oophorectomy) may be considered [1].

The prognosis of pulmonary leiomyoma and BML is generally favorable, with most patients demonstrating indolent disease and low risk of progression or recurrence following complete resection [1].

A brief review of the literature demonstrates that pulmonary benign metastasizing leiomyoma (PBML) most commonly presents as incidentally detected multiple bilateral pulmonary nodules in women with a prior history of uterine leiomyoma or hysterectomy. Endobronchial involvement, however, is exceptionally uncommon, with only isolated case reports described in the literature [5].

Large case series by Fan et al. involving 23 patients with PBML showed that the predominant radiological pattern was multiple well-circumscribed pulmonary nodules, while endobronchial growth was not a typical manifestation. Most patients were asymptomatic or presented with nonspecific respiratory symptoms such as cough or dyspnea [6].

Similarly, Dai et al. [7] and Taftaf et al. [8] reported that PBML usually manifests as parenchymal lung nodules discovered years after hysterectomy for uterine leiomyoma, often creating a diagnostic dilemma by mimicking metastatic malignancy. Cavitary and cystic lesions have also been rarely described.

In contrast, endobronchial BML is exceedingly rare and may clinically resemble primary bronchogenic carcinoma or endobronchial carcinoid due to symptoms related to airway obstruction, including cough, wheeze, recurrent pneumonia, lobar collapse, or hemoptysis. Bronchoscopic evaluation in such cases typically reveals a polypoidal or obstructing endobronchial lesion, and definitive diagnosis relies on histopathological examination with immunohistochemistry demonstrating benign spindle cell proliferation positive for smooth muscle markers (SMA and desmin) and hormone receptors (ER/PR) with low proliferative index [2,9,10].

The present case adds to the limited literature on endobronchial presentations of BML and highlights the importance of considering this rare entity in women presenting with endobronchial lesions and a prior history of uterine leiomyoma, thereby avoiding misdiagnosis as primary or metastatic pulmonary malignancy.

## Conclusions

Pulmonary leiomyoma should be considered in the differential diagnosis of lung lesions, particularly in women with a history of uterine leiomyoma and hysterectomy. The distinction from malignancy is challenging preoperatively, and histopathological examination remains essential for definitive diagnosis. Surgical resection is both diagnostic and curative in most cases, with an excellent prognosis.
